# Awareness of Nuclear Medicine Physicians in Romania Regarding the Diagnostic of Cardiac Amyloidosis—A Survey-Based Study

**DOI:** 10.3390/diagnostics12020556

**Published:** 2022-02-21

**Authors:** Claudiu Stan, Raluca Mititelu, Robert Daniel Adam, Ruxandra Jurcuţ

**Affiliations:** 1 Department of Nuclear Medicine and Ultrasonography, Fundeni Clinical Institute, 022328 Bucharest, Romania; 2 Department of Cardiothoracic Pathology, Carol Davila University of Medicine and Pharmacy, 020021 Bucharest, Romania; robert.adam@drd.umfcd.ro (R.D.A.); ruxandra.jurcut@umfcd.ro (R.J.); 3 Department of Nuclear Medicine, Carol Davila Central University Emergency Military Hospital, 010825 Bucharest, Romania; ralunuclear@yahoo.com; 4 Expert Center for Rare Genetic Cardiovascular Diseases, Department of Cardiology, C.C. Iliescu Emergency Institute for Cardiovascular Diseases, 022328 Bucharest, Romania

**Keywords:** amyloidosis, nuclear medicine, awareness, survey, ATTR

## Abstract

Amyloidosis is a heterogeneous group of diseases caused by the extracellular deposition of amyloid insoluble fibrils in multiple organs, resulting in various clinical manifestations. Cardiac amyloidosis (CA) occurs mainly in primary light-chain (AL) amyloidosis, hereditary transthyretin (ATTRv) amyloidosis and senile or wild-type transthyretin (ATTRwt) amyloidosis. Knowing that myocardial uptake at bone scintigraphy is an essential step in the ATTR-CA diagnostic algorithm, the level of awareness among nuclear medicine physicians (NMPs) using bone tracer scintigraphy is of great importance. The objective of the study was to evaluate NMPs’ awareness of scintigraphy with bisphosphonates for the detection of CA. We conducted an online survey among NMPs from Romania to assess their current awareness and state of knowledge of nuclear techniques used in CA. Among the total 65 Romanian NMPs, 35 (53%) responded to this questionnaire. Approximately three-quarters of participants (74%) found a diffuse accumulation of bisphosphonates in the heart on scintigraphy performed for bone pathology as an incidental discovery. Detection of myocardial uptake of 99mTc-labeled bisphosphonates on scintigraphy suggests CA-AL for 3% of participants and for 9% of respondents, the appearance is of uncertain cardiac amyloidosis, while 5% of participants observed cardiac uptake but did not report it as CA. Even if more than half of those who responded to this survey (54%) found abnormal cardiac uptake and interpreted it as CA-ATTR, only 14% contacted the referring physician to draw attention to the incidental discovery to refer the patient to a specialist in rare genetic cardiomyopathy. Regarding the knowledge about the categories of bisphosphonates recommended in the diagnosis of CA-ATTR, 54% answered inadequately that methylene diphosphonate (MDP) could be used. Romanian nuclear physicians are partially familiar with CA diagnosis by scintigraphy, but its diagnostic potential and standardization, recommended radiotracers and acquisition times and interpretation algorithms are known in varying proportions. Therefore, there is a need to enhance knowledge through continuing medical education programs in order to standardize the protocols for the acquisition, processing and interpretation of bisphosphonate scintigraphy for the detection of cardiac ATTR amyloidosis.

## 1. Introduction

Amyloidosis is a disease produced by the deposition of specific amyloid fibril proteins in different organs and tissues. Amyloid is recognized microscopically by its amorphous structure, an affinity for the Congo red dye and its increased birefringence under polarized light. According to the International Society of Amyloidosis Nomenclature Committee, the number of mature proteins responsible for amyloidosis is 18 proteins for systemic amyloidosis and 22 for localized forms; even some proteins can appear as systemic and localized amyloid deposits [1]. 

Of the 36 human amyloid fibrillar proteins currently discovered, only 9 can involve the myocardium and cause significant heart disease. However, more than 98% of amyloid cardiomyopathies are due to light chain monoclonal immunoglobulins (AL) and fibrilar transthyretin (ATTR). Other types of cardiac involvement (AApoAI, AApoAII, AApoAIV, Aβ2M, AFib, AGel) and cardiac amyloidosis secondary to chronic inflammatory and infectious diseases (AA) are very rare in medical practice [1,2]. 

ATTR cardiac amyloidosis is the second most common form of CA after cardiac involvement in AL amyloidosis. The two types of ATTR cardiac amyloidosis (ATTR-CA) are the wild type (ATTRwt-CA), previously called senile, and the hereditary or variant form (ATTRv-CA). Having more than 130 genetic variants detected, ATTRv-CA is a rare autosomal dominant condition caused by mutations in the transthyretin gene [3]. ATTRwt amyloidosis has 100% heart involvement, with a median survival from diagnosis of 57 months, while in ATTRv amyloidosis, the frequency of cardiac localization varies between 30–100%, and the median survival is 31 months, depending on the mutation [2]. The usual extracardiac signs in ATTRwt amyloidosis are bilateral carpal tunnel syndrome, lumbar spinal stenosis, and biceps tendon rupture. Other extracardiac signs in ATTRv amyloidosis depend on the tissue location of amyloid fibrillar proteins: polyneuropathy, orthostatic hypotension, vitreous opacities, and gastrointestinal manifestations [2]. Recent studies have shown an increased prevalence of up to 15% of ATTRwt cardiac amyloidosis in older adults with heart failure with preserved ejection fraction (HFpEF) or in severe aortic stenosis [4,5,6]. 

The diagnostic criteria for CA are divided into invasive and noninvasive diagnostic techniques. In the presence of amyloid fibrillar proteins identified on endomyocardial biopsy samples using Congo red staining, cardiac amyloidosis can be confirmed. Identification of the type of amyloid is performed in clinical practice by immunohistochemistry or immunoelectron microscopy. Along with the characteristic features from echocardiography or cardiac magnetic resonance imaging, the presence of amyloid deposits at extracardiac biopsy completes the invasive diagnosis of cardiac amyloidosis [2]. However, the diagnostic yield of fat aspirate for amyloidosis is much lower for ATTRwt (15%) and ATTRv (50%) than for AL amyloidosis [7]. 

While invasive criteria apply to all cases of cardiac amyloidosis, extracardiac biopsy sensitivity is low for ATTR, and endomyocardial biopsy has a high-risk profile. The noninvasive criteria for the diagnosis of ATTR-CA consist of positive bisphosphonate scintigraphy and absence of monoclonal dyscrasia, along with typical findings on echocardiography and CMR [8]. A Perugini visual score of 2 or 3, corresponding to a degree of myocardial uptake, on bisphosphonate scintigraphy with 99mTc-pyrophosphate (PYP), 99mTc-3,3-diphosphono-1,2-propanodicarboxylic acid (DPD) or 99mTc-hydroxymethylene diphosphonate (HMDP), has already been validated by multiple clinical trials for the positive diagnosis of ATTR-CA [9,10]. Plasma cell proliferative diseases can be ruled out with a sensitivity of 100% by the absence of fibrillar protein precursors in serum and urine using the following tests: serum and urinary protein electrophoresis with immunofixation (sIFE, uIFE) and detection of serum-free, light chains with kappa/lambda ratio (sFLC κ/λ) [11]. 

With the recent indication for TTR stabilizers (tafamidis) as effective therapies for reducing ATTR-CA all-cause mortality and cardiovascular-related hospitalizations [7,12], an early diagnosis is essential for instituting the earliest effective therapy, casting the importance of awareness of diagnostic methods.

We, therefore, conducted the current survey among nuclear medicine physicians in Romania to evaluate their awareness of the diagnostic features and forms of cardiac amyloidosis and how it is reflected in their daily medical practice.

## 2. Materials and Methods

An online survey was conducted among nuclear medicine physicians (NMPs) from all hospitals and health centers in Romania during a 4-month interval between 1 October 2020 and 31 January 2021. It consisted of 31 questions with single or multiple answers developed by the authors, which included a general part (working place, level of medical expertise in general and nuclear cardiology in particular) and a special section regarding diagnostic features in CA. 

The questionnaire was developed based on the authors’ experience, who are part of a multidisciplinary diagnostic team for CA in an Expert Center for Cardiac Rare Disease and considering international consensus and up-to-date literature, as well as based on recommendations for surveys [13,14,15]. 

The complete copy of the online questionnaire is available on “Supplementary Materials”. The survey was uploaded to a platform dedicated to online questionnaires and was distributed to NMPs who were or were not members of the Romanian Society of Nuclear Medicine and Molecular Imaging (SRMNIM) by e-mail or messaging applications. 

The specialty of nuclear medicine in Romania includes about 65 NMPs, of which 15 are in different training stages. In total, 35 NMPs (53%) answered the questionnaire. All participants could answer the questionnaire only once. Participation in the questionnaire was anonymous and voluntary, and participants were informed that the authors planned to publish the results, so ethical approval was not required.

After the end of the 4-month period, the data were collected, and the results were calculated as a percentage of the total number of responses.

## 3. Results

### 3.1. Survey Participants

**Figure 1:**
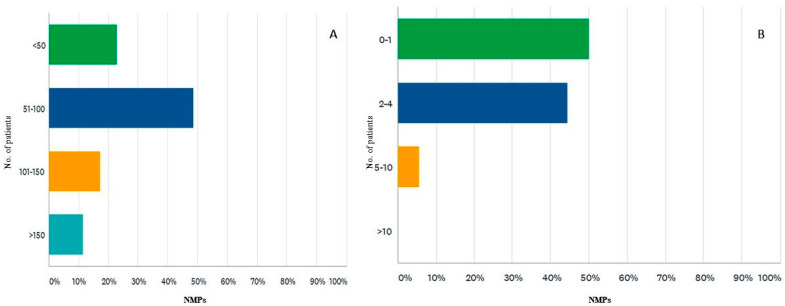
(**A**) Number of patients investigated by scintigraphy for all indications, routinely each month by nuclear medicine physicians. (**B**) Number of patients referred each month for bisphosphonate scan and investigated by nuclear medicine physicians involved in the diagnosis of CA. NMPs-nuclear medicine physician.

Of the 35 survey participants, 85% practiced in the 3 largest Romanian university centers: 45% in the country’s capital (Bucharest), 28% from Iasi and 11% from Cluj. Regarding training, out of the three levels of training in Romania, 62% were senior NMPs (highest level), 22% were specialized NMPs (intermediate level), and 14% were NMPs in training. This distribution is like the career levels distribution of the members of the National Society for Nuclear Medicine in Romania. Of the 35 participants who responded to this survey, 80% work in a university hospital. Moreover, the work environment was reported as a public hospital (82%), private medical center (only 17%), and both in a public hospital as well as in a private one (25%). Currently, each month, the number of patients investigated by conventional nuclear medicine techniques (e.g., bone scintigraphy BS, myocardial perfusion scintigraphy and so on) exceeds 150 (only for 11% of respondents), falling within the range of 101–150 (for 17%), between 51–100 (for most of NMPs, 48%), and under 50 (22% of NMPs) (Figure 1A). 

**Figure 2:**
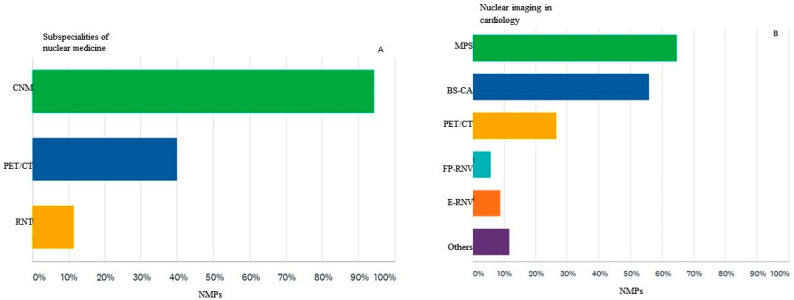
(**A**) Number of nuclear medicine physicians (NMPs) participating in the survey who practice the subspecialties of nuclear medicine, represented by conventional nuclear medicine (CNM), positron emission tomography with computer tomography (PET/CT) and radionuclide therapy (RNT). (**B**) The main types of radionuclide investigations of nuclear cardiology performed by survey participants MPS = myocardial perfusion scintigraphy, BS-CA = bisphosphonate scintigraphy for detecting cardiac amyloidosis, PET/CT = positron emission tomography with computer tomography, FP-RNV = first-pass radionuclide ventriculography, E-RNV = equilibrium radionuclide ventriculography.

The main subspecialties of nuclear medicine are represented by conventional nuclear medicine (CNM), which uses planar scintigraphic techniques or SPECT with or without hybrid techniques (SPECT/CT), in which most participants work, 94%, PET/CT in which activate 40% of the participants and the radionuclide therapy (RNT) represented by 11% of the respondents (Figure 2A). 

Regarding the main types of nuclear cardiology imaging investigations performed, of the 34 participants who answered this question, the majority (64%) report results of myocardial perfusion scintigraphy (MPS) and just over half of them (55%) bisphosphonate scintigraphy for detecting cardiac amyloidosis (BS-CA). About a quarter of them (26%) use cardiac PET /CT mainly for the diagnosis of infective endocarditis and vasculitis. Equilibrium radionuclide ventriculography (E-RNV) and the first pass study (FP-RNV) are performed by 8% and 6%, respectively, while 11% of them perform other types of imaging investigations of nuclear cardiology (Figure 2B).

### 3.2. Experience with Cardiac Amyloidosis

The approximate number of scintigraphic investigations currently performed to detect cardiac amyloidosis performed in a month by survey participants is one or no investigation/month for 50% NMPs, between 2 and 4 in 44% NMPs; and only 1 participant performs more than 5 investigations/month (Figure 1B). Most survey participants (82%) are interested in cardiac amyloidosis and have participated in conferences or courses dedicated to applications of nuclear medicine in CA. However, most of the participants (65%) are not involved in the multidisciplinary team for the diagnosis of CA in the institution where they work. 

**Figure 3:**
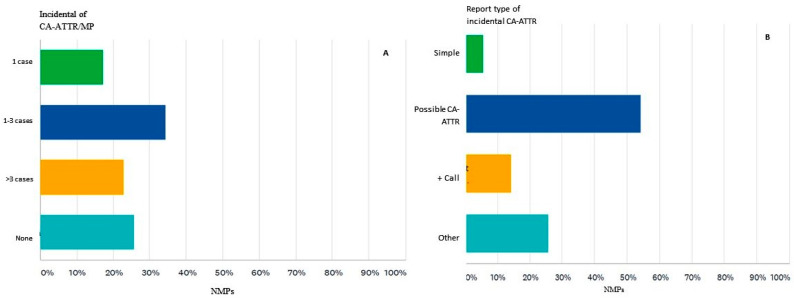
(**A**) The number of cases of diffuse increased myocardial uptake on bisphosphonate scintigraphy with clinical indication other than CA-ATTR, incidentally encountered throughout the medical profession (MP). (**B**). How the survey participants reported the incidental discovery of pathological myocardial uptake on bisphosphonate scintigraphy Simple = reported, without further comment on the possible cause of CA-ATTR, Possible CA-ATTR = reported, with mention of the possible cause of myocardial uptake of bisphosphonate, +Call = possible CA-ATTR and contacted the referring doctor, Other = no diffuse increased uptake of the myocardium was observed on bisphosphonate scintigraphy in medical practice, NMPs = nuclear medicine physicians.

Nearly three-quarters of the participants (74%) found an incidental diffuse accumulation in the cardiac projection in a bisphosphonate scintigraphic investigation performed for an indication other than cardiac amyloidosis (e.g., scintigraphy performed for suspected bone metastases) (Figure 3A). If the participants found an increased and diffuse cardiac uptake in a bisphosphonate scintigraphy investigation, they proceeded in various ways. Most participants (54%) reported abnormal cardiac uptake and interpreted it as possible CA-ATTR. However, 5% of the participants noted cardiac uptake but did not interpret it as CA. Further, it appears that they did not find it in 25%, so they did not report the existence of bisphosphonate myocardial uptake. Only 14% of participants contacted the referring physician to draw attention to the incidental findings and to send the patient to the specialized multidisciplinary team (Figure 3B).

### 3.3. Cardiac Amyloidosis—Imaging Awareness

**Figure 4:**
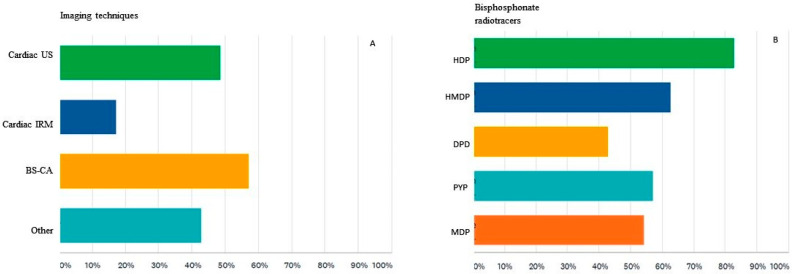
(**A**) Imaging techniques used for the diagnosis of patients with CA in the respondents’ institution or in the one they work with. Cardiac US = ecocardiography, Cardiac IRM = cardiac magnetic resonance imaging, Bone Scan = bisphosphonate scintigraphy for CA detection, Other = survey participants do not work or collaborate with a cardiology department. (**B**) 99mTc-labeled bisphosphonated radiotracers used in CA diagnosis. HDP = hydroxyethylene diphosphonate, HMDP = hydroxymethylene diphosphonate, DPD = 2,3-dicarboxypropane-1,1-diphosphonate, PYP = sodium pyrophosphate, MDP = methylene diphosphonate, NMPs = nuclear medicine physicians.

Imaging tests used to diagnose patients with suspected CA at the institution where they work were considered by survey participants to be the following: echocardiography in 48% of cases, cardiac magnetic resonance imaging in 17% of cases and bisphosphonate scintigraphy in 57% of cases. There were 42% of respondents that said cardiac imaging is recommended in another medical unit (Figure 4A). 

When verifying their knowledge regarding the 99mTc-labeled bisphosphonate tracers used for detecting cardiac amyloidosis, the answers were divided as follows: 82% have chosen hydroxyethylene diphosphonate (HDP), 62% chose hydroxymethylene diphosphonate (HMDP), 42% chose 2,3-dicarboxypropane-1,1-diphosphonate (DPD), 57% chose sodium pyrophosphate (PYP), and over half of them, or 54%, chose methylene diphosphonate (MDP) (Figure 4B).

**Figure 5:**
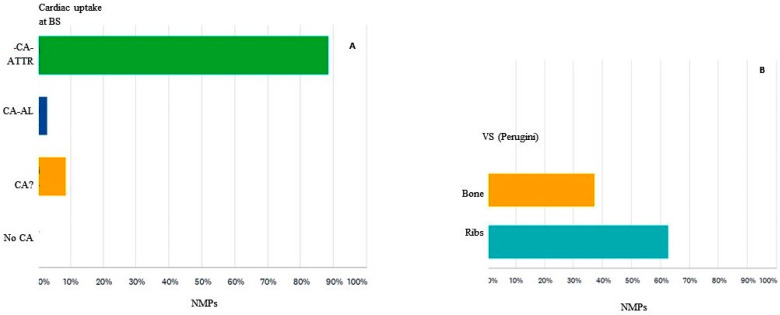
(**A**) Interpretation of survey participants of scintigraphy in which bisphosphonates accumulate in the myocardium. CA-ATTR = cardiac amyloidosis with transthyretin, CA-AL = cardiac amyloidosis with light chains, CA? = cardiac amyloidosis of uncertain nature, No CA = the appearance is not related to cardiac amyloidosis, M = myocardium, BS = bone scan. (**B**) Semi-quantitative analysis score (Perugini) of cardiac uptake of 99mTc-bisphosphonate compared to uptake of the ribs, VS = visual score, Bone = VS related to bone, Ribs = VS related to ribs, NMPs = nuclear medicine physician, BS = bone scan.

The detection of myocardial uptake of 99mTc-labeled bisphosphonates on scintigraphy suggests for most participants (88%) CA with fibrillar TTR protein (CA-ATTR). However, for 3% of participants, the aspect described above suggests light chain amyloidosis (CA-AL), and for 9% of respondents, the appearance is of uncertain CA for which an additional assessment is needed to specify the type of amyloid (Figure 5A). A score of semi-quantitative analysis (Perugini) of cardiac uptake of 99mTc-bisphosphonate refers in the opinion of participants to the degree of uptake of the radiotracer in relation to the uptake of bones for 37% of them, while for almost 63%, it represents the degree of uptake of the radiotracer in relation to the uptake of the ribs (Figure 5B).

Of note, the interest in creating teaching materials on systemic amyloidosis and organizing scientific events in this field is manifested in more than three quarters (77%) of participants.

### 3.4. Cardiac Amyloidosis in the Nuclear Medicine Laboratory 

The distribution of responses regarding the use of bisphosphonate radiotracers for CA detection by the responding NMPs was relatively symmetrical, with almost 49% of them giving an affirmative answer. We further report on the answers of these 18 NMPs who perform bisphosphonate scintigraphy. Among them, 50% describe the institution where they work as a reference center for amyloidosis. 

**Figure 6:**
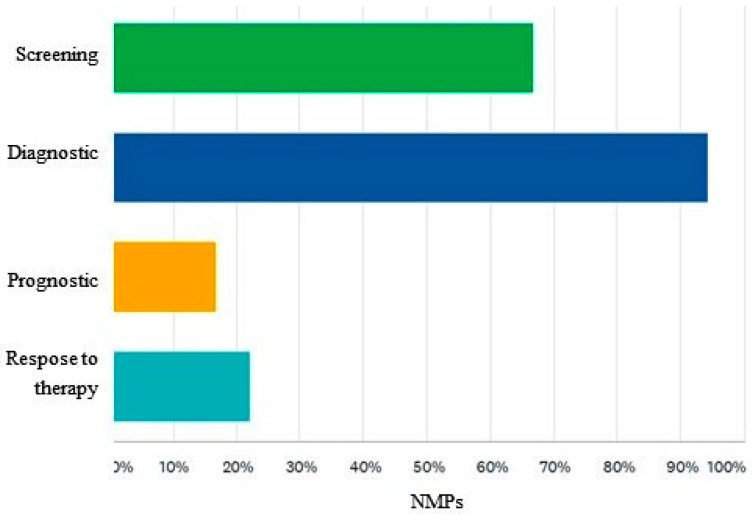
Perception of bisphosphonate scintigraphy clinical indications in amyloidosis management among NMPs. NMPs = nuclear medicine physicians.

Among these physicians, the perception regarding the clinical indications of scintigraphy for the detection of CA is the following: 66% of answering NMPs would use it for the screening of the disease, 94% for the diagnosis, 16% for the prognosis of the disease, and 22% for the evaluation of the response to therapy (Figure 6). 

**Figure 7:**
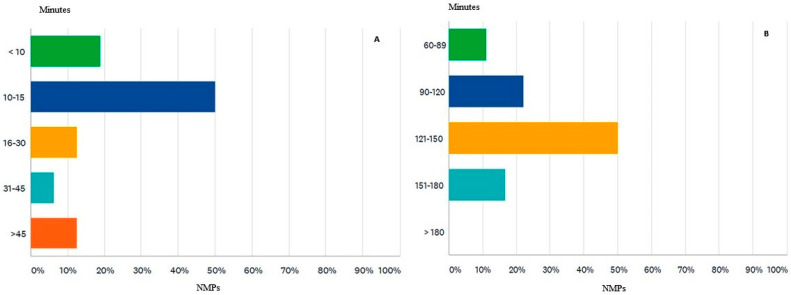
Time interval between the administration of 99mTc-bisphosphonate for the early (**A**) and late (**B**) acquisition.

When asked about the average recommended dose for bisphosphonate scintigraphy for CA detection, the majority (77%) answered that it is in the range of 555–740 MBq (megabecquerel), and 11% considered the optimal dose between 370–554 MBq. Regarding the time interval between the administration of 99mTc-bisphosphonate and the acquisition of scintigraphy data, all participants use late data acquisition (between 1–3 h), while 38% of participants also use early acquisition (10–15 min) (Figure 7A). The answers on the optimal time interval for the late acquisition of data have a rather varied distribution as follows: 50% consider 121–150 min, followed by those (22%) who appreciate the appropriate interval between 90–120 min, 16% apply the interval 151–180 min, and finally, 11% use the interval 60–89 min to detect the distribution of the radiotracer (Figure 7B).

**Figure 8:**
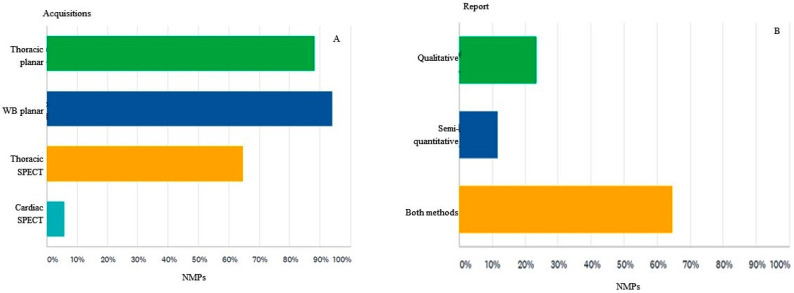
(**A**). Image acquisition protocol, part of the imaging protocol in CA-ATTR diagnosis. Thoracic planar = Acquisition of image in the same plane in antero-posterior and oblique incidents. WB planar = Incidents in the same plane anterior and posterior whole body (vertex-soles). SPECT = Single photon-emission computed tomography at the thorax. Cardiac SPECT = Single photon-emission computed tomography only at the level of the heart. (**B**). Reporting the uptake of 99mTc-bisphosphonate in the myocardium.

The imaging protocol in the scintigraphic diagnosis of CA is represented among the survey participants by the following types of acquisition: whole-body planar (94%), thoracic planar (88%), thoracic SPECT (64%) and only cardiac SPECT (5%) (Figure 8A). 

Visual or qualitative analysis of bisphosphonate scintigraphy for describing the appearance of the radiotracer uptake at the level of the heart, such as no uptake, focal uptake, diffuse uptake, focal uptake superimposed on the diffuse one, is used by all participants (100%) involved in the imaging diagnosis of CA. Survey respondents interpret bisphosphonate scintigraphy quite differently: 23% only qualitatively, in which CA is present or absent, 11% only quantitative or semi-quantitative, through various reporting methods, and 64% use both methods in the scintigraphy interpretation (Figure 8B). 

In the semi-quantitative interpretation of cardiac uptake, the Perugini visual score (VS 0–3) is used by 88% of participants who currently use scintigraphy for CA detection. In the opinion of the respondents, the diagnostic significance attributed to the visual score Perugini 1 (VS 1) makes the existence of a CA-ATTR unlikely for 82% of the respondents, while being considered diagnostic for CA-AL in 17%. The quantification of the uptake of the 99mTc-bisphosphonate radiotracer from the heart, related to the contralateral region or H/CL, as part of the interpretation of the scintigraphic examination in CA, is used by over half of the participants who are involved in the diagnosis of CA (52%).

## 4. Discussion

The present study reports the results of the first national survey conducted in Romania regarding current awareness and knowledge on CA and nuclear techniques among NMPs. Such data are sparse in the literature, even though we conducted it during a time frame when the increasing knowledge and interest regarding amyloidosis in general and CA, in particular, is driven by the emergence of diagnostic and therapeutic options for severe diseases. 

Current evidence and a need to standardize the most effective diagnostic and therapeutic methods led to the development of “A Scientific Statement from the American Heart Association” in July 2020 [16] and “A Position Statement from the ESC Working Group on Myocardial and Pericardial Diseases” in April 2021 [2]. Several national cardiology societies developed guidelines for the evaluations and management of CA in the last two years [17,18,19]. Dorbala et al. developed in December 2019 the “Expert consensus recommendations for multimodality imaging in cardiac amyloidosis” in two parts: “Evidence Base and Standardized Methods of Imaging” and “Diagnostic Criteria and Appropriate Utilization”, respectively, representing complete guidelines for current imaging practice in CA [9,10], with a more recent Addendum detailing several acquisitions and reporting rules [20]. The Romanian Society of Nuclear Medicine and Molecular Imaging (SRMNIM) has translated in June 2021 the European Nuclear Medicine Guide—a joint publication by EANM and UEMS/EBNM, to be included in the training curriculum for physicians in training [21].

However, this first survey among Romanian NMPs primarily reflects several gaps in knowledge about the diagnostic use of BS and imaging interpretation in ATTR-CA. We believe that this varied distribution of NMPs responses is an opportunity to increase continuing medical education hours on the contribution of bisphosphonate scintigraphy in CA detection. Additionally, 77% of participants are interested in creating teaching materials and organizing scientific events related to systemic amyloidosis. Currently, in Romania, BS with bisphosphonates is by far the most used diagnostic imaging method that belongs to CNM, and this is an advantage in both screening and diagnosing patients with CA-ATTR, but also in discovering incidental cases in patients with another indication by performing BS. Several measures could assist in improving knowledge in this rare clinical condition: involving NMPs in an “amyloidosis team,” efficient reporting of CA-ATTR incidentalomas, and knowledge of diagnostic guidelines and protocols of nuclear imaging.

Another study related the knowledge to the diagnosis and therapy of CA among Romanian cardiologists was also conducted by Adam et al. The study’s findings highlight that the Romanian cardiologists are partially aware of CA, but several misconceptions about the diagnostic suspicion of CA and therapy, emphasize the need to update the knowledge related to this pathological condition [22].

### 4.1. Involvement of NMPs in the “Amyloidosis Team”

Only 35% of the participants are involved in the nuclear diagnosis of CA-ATTR, and 44% of them perform between 2 and 4 BS per month for the diagnosis of CA. Most NMPs (65%) are not part of an “amyloidosis team” that includes at least one cardiologist, a hematologist, a neurologist, a pathologist, and a radiologist to perform cardiac MRI.

The concept of the amyloidosis team was presented in the recent ESC position statement on CA diagnosis and therapy, which mentioned that specific treatment in cardiac AL amyloidosis should be undertaken by multidisciplinary teams involving oncohaematology and cardiology specialists [2]. While Dorbala et al. published a document on the use of multimodality imaging in CA by a panel of multidisciplinary experts, they did not refer to the need of including cardiac imaging specialists from the various techniques in a larger amyloidosis team. We know now that several particular situations exist in the use of nuclear medicine for CA. Recent studies demonstrated suboptimal sensitivity of DPD scintigraphy in patients carrying the early-onset Val50Met (formerly Val30Met) variant and other genotypes like Phe64Leu. Therefore, the accuracy of the technique may be influenced by the ATTRv genetic variant [23,24]. Moreover, a Perrugini score of 1 and even higher can be found in CA-AL, and this should be excluded using hematological tests [7,23,25]. Other examples reflecting the importance of a multidisciplinary amyloidosis team are echocardiography or cardiac MRI highly suggestive of CA and negative BS, BS positive and abnormal FLC (free light chain) [9,25], and BS incidentaloma positive for CA-ATTR.

Therefore, with the current knowledge that CA is not a rare disease (especially ATTRwt) and is part of systemic involvement, the concept of amyloidosis team should be taken further because involving NMPs in a multidisciplinary expert panel during the diagnosis and follow-up of patients will enhance expertise and lead to an earlier and more precise diagnosis for the patients.

### 4.2. CA-ATTR Incidentalomas in Bone Scan

Probably one of the situations we encounter quite often in medical imaging in general and in bisphosphonate scintigraphy with an oncological indication, in particular, is represented by incidentalomas, i.e., the accidental discovery of pathology, in this case, of CA-ATTR. Bone scintigraphy with oncological indication represents most CNM investigations in Romania, and NMPs can encounter various imaging findings, e.g., bone metabolic diseases (Paget’s disease, renal osteodystrophy with hyperparathyroidism) or the more frequent appearance of bone trauma. There is a varied experience with incidental findings in bisphosphonate scintigraphy: while 25% of the responding NMPs did not encounter in daily medical practice any case of diffuse cardiac uptake in the myocardium at a BS performed for an indication other than the suspicion of CA-ATTR, another 22% encountered more than three cases during their professional career. Of the 75% of respondents who encountered at least one case of a positive BS for CA, only 14% contacted the referring physician to draw attention to the incidental discovery, while 5% did not interpret the scintigraphy aspect as possible coexistence of CA. Given that CA-ATTR is a rare disease, with a prevalence often underestimated even by cardiologists [26], other specialties that refer the patient for bone scintigraphy (oncology, rheumatology, urology, surgery, etc.) could overlook such a diagnosis, with direct consequences on the possibility of treatment of the patient. If not aware of its significance, myocardial uptake during bisphosphonate scintigraphy might be overlooked without reporting the suspicion of CA-ATTR. 

### 4.3. Significance of Cardiac Uptake and Types of Bisphosphonate Radiotracer

While 88% of responders correctly link the myocardial uptake during bisphosphonate scintigraphy to CA-ATTR, more than 10% of respondents stated that the reason for myocardial uptake of 99Tc-labeled bisphosphonates could be a CA-AL or a CA with fibrillar proteins of uncertain nature that require further investigation to determine the type of amyloid. Moreover, the semi-quantitative analysis score (visual score VS Perugini) refers in the opinion of 32% of the participants to the degree of uptake of bisphosphonate in the heart in relation to bone uptake and not as specified in Dorbala et al. in the Expert Consensus Recommendation, with the one at the rib level [9]. 

One of the most inconsistent responses was related to the use of 99mTc-bisphosphonate types, in which more than half of the participants answered that methylene diphosphonate (MDP) could be used for CA-ATTR detection. The tracers 99mTc-MDP and 99mTc-aprotinin are not recommended for imaging cardiac amyloidosis [16]. Recommended radiotracers based on 99mTc-bisphosphonates for the diagnosis of ATTR amyloidosis are 99mTc-pyrophosphate (99mTc-PYP), 99mTc-3,3-diphosphono-1,2-propanodicarboxylic acid (99mTc-DPD) and 99mTc-hydroxymethylenediphosphonate (99mTc-HMDP) [9].

Regarding the use of 99mTc-bisphosphonate BS results, 16% answered that scintigraphy has prognostic value, while 22% believe that it is useful in evaluating the response to therapy. Castano et al. highlighted in a multicenter study of planar Technetium 99m Pyrophosphate (PYP) Cardiac Imaging that a H/CL ratio of 1.6 or greater was associated with worse survival among patients with ATTR cardiac amyloidosis [27]. The association of 99mTc-PYP uptake with interventricular septum thickness or NYHA classes of heart failure or NT-proBNP levels improves prognostic risk stratification. Although the semi-quantitative evaluations of the H/WB ratio for 99mTc-DPD and the H/CL ratio for 99mTc-PYP could identify the increased risk for major adverse cardiac events, the visual gradient of 99mTc-HMDP/DPD did not prove to be an independent predictor of outcome [28]. However, monitoring CA-ATTR using nuclear techniques lacks evidence. In the recently published ESC position statement on CA [2], Garcia-Pavia et al. stated that the value of nuclear techniques in assessing the progression of ATTR-CM is not fully elucidated, and therefore, the expert group did not make a recommendation for their use in disease monitoring.

### 4.4. Practical Aspect in the Nuclear Medicine Laboratory

The average dose administered for 99mTc-bisphosphonate is in the range of 370–740 MBq, according to recommendations for standardized acquisition of 99mTc-PYP/DPD/HMDP for CA [9]. More than three-quarters of our survey participants use the upper half of the reference range (555–740 MBq), while only 11% use the lower recommended doses (370–554 MBq) for an optimal diagnosis of CA. The remaining 11% do not know the recommended dose range. 

Regarding the early acquisition of scintigraphic data, 38% of the participants, using 99mTc-bisphosphonate scintigraphy for detecting cardiac amyloidosis, gave an affirmative answer, but when asked about the time between the administration of the radiotracer and the early acquisition, the distribution of answers was quite different. These results indicate a lack of knowledge of the results obtained through the early acquisition of scintigraphic data and their use in current practice. Early whole-body counts are used to represent the injected activity. Whole-body (WB) retention is calculated by subtraction from early to late WB (corrected for disintegration and scanning speed, decreasing bladder and kidney activity). Heart retention (HR) is obtained by comparing decay-corrected counts of the heart in late images with counts in early whole-body images. The H/WB ratio refers to the heart and whole-body late counts ratio. These are semi-quantitative analyses used especially in clinical trials to accurately assess the dose administered, cardiac retention and whole-body distribution of the radiotracer [29].

According to the same consensus [9], the late acquisition of scintigraphic data after the administration of 99mTc-bisphosphonates is performed within 1–3 h. More precisely, for 99mTc-PYP, the recommended waiting time is 1 h and optionally 3 h if there is a risk of false results (for example, the persistence of the excess radiotracer in the blood pool on the images at 1 h), while for 99mTc-HMDP/DPD recommendation is 2 or 3 h after injection of the radiotracer. Meanwhile, in August 2021, Dorbala et al. published an Addendum [20] to the previous consensus [9], stating that the recommended time interval between 99mTc-PYP/DPD/HMDP injection and acquisition is 2–3 h. Optionally for 99mTc-PYP, scintigraphic data acquisition is maintained at 1-h and rescan at 3-h in case of inadequate imaging (excess blood pool noted at 1-h) Noting that in Romania, 99mTc-HDP is most largely bisphosphonate radiotracer used, of all survey participants who are involved in the diagnosis of CA by BS, we notice a varied distribution of answers, so almost 67% of them prefer late acquisitions in 2–3 h, while 22% make an intermediate acquisition (90–120 min) not specified in the consensus. Only 11% prefer to obtain scintigraphic data between 60 and 90 min. 

Thoracic SPECT was reported to be performed by only 64% of those who perform scintigraphy to detect cardiac amyloidosis. Dorbala et al. recommended [9] the use of SPECT acquisition for all patients in whom planar imaging is positive for the standardized acquisition of 99mTc-PYP/DPD/HMDP for cardiac amyloidosis. In the last position statement of the ESC Working Group [2], the authors emphasize the need to perform SPECT acquisition for all patients in whom there is a risk of false-positive or negative results. In the recent Addendum [20], Dorbala et al. introduced cardiac SPECT as required for all patients with suspected ATTR-CA. In addition, 1-h planar imaging is no longer recommended, being replaced with 2-h or 3-h imaging for all recommended bisphosphonates (99mTc-PYP/DPD/HMDP). 

The results of 99mTc-bisphosphonate scintigraphy for CA detection include a two-step approach: visual interpretation for the positive diagnosis of CA-ATTR and semi-quantitative grading. Nearly 65% of survey participants correctly use both approaches in assessing BS. The rest of the participants evaluate the scintigraphy either only by qualitative visual interpretation (23%) or only by semi-quantitative approach (12%). Processing of anterior planar images 2–3 h after 99mTc-PYP/DPD/HMDP administration involves choosing a circular or elliptical area to circumscribe the heart (H) to the left of the sternum, without overlapping it or the left lung. The same surface overlaps in the mirror in the contralateral region (CL), right parasternally, for the background, and the H/CL ratio is calculated as the ratio of the number of counts from the two described areas. A H/CL ≥ 1.5 on 99mTc-PYP/DPD/HMDP acquisition can distinguish with a sensitivity of 97% and a specificity of 100% CA-ATTR from that of AL amyloidosis. The exclusion of AL amyloidosis should always be done with serum/urine immunofixation and a serum-free light-chains assay in all patients with suspected amyloidosis [20,29,30]. 

Regarding the diagnostic significance of the G1 visual score, for 82% of participants, the existence of a CA-ATTR is unlikely. For the remaining 18%, G1 is suggestive for the diagnosis of light chain amyloidosis. Late anterior planar images, 2–3 h after 99mTc-bisphosphonate injection, allow the assessment of relative myocardial uptake, related to ribs and uptake grading in 4 scales: G0-without myocardial uptake with normal ribs uptake, G1-myocardial uptake less than ribs uptake, G2-myocardial uptake equal to ribs uptake and G3-myocardial uptake higher than ribs with mild/absent ribs uptake. The diagnosis of CA-ATTR is consistent with a relative uptake of the radiotracer in the myocardium with G2 or G3 grading. Additionally, it is necessary to exclude systemic AL amyloidosis because in over 20% of positive scintigraphy diagnostics for CA-ATTR, cardiomyopathy with light chain fibrillar proteins may exist or coexist [9,20]. Moreover, in 40% of CA-AL patients, myocardial uptake can be found, so it always requires the exclusion of monoclonal processes [7,9,23]. Of those who use the semi-quantitative approach to interpret scintigraphy, the majority (88%) use visual grading (Perugini), while the H/CL ratio approach is used by 53% of participants. 

### 4.5. Comparative Value of BS Versus Other Investigations

The specificity and positive predictive value of bisphosphonate scintigraphy (99mTc-PYP/DPD/HMDP) for grade 2 or 3 myocardial uptake for ATTR-CA, in the absence of detectable monoclonal proteins in serum and urine (sIFE, uIFE and sFLC κ/λ) is 100% [8]. False-positive or false-negative BS results could appear in some conditions. False-positive results for ATTR-CA may occur in: AL-amyloidosis, hydroxycloquine-induced restrictive cardiomyopathy, rare variants of CA associated with chronic kidney disease (AapoAI, AApoAII, ApoAIV or Aβ2M amyloidosis) or in radiotracer persistence in the blood pool. We may encounter false-negative results for ATTR-CA in the following circumstances: rib fractures or valvular calcifications, recent myocardial infarction, genetic variants with reduced sensitivity to bisphosphonate scintigraphy (Phe64Leu [23], Phe84Leu, Ser97Tyr [2]), a mild form of amyloidosis or noncompliance with acquisition types for scintigraphy [2]. 

There are several advantages regarding BS as compared to other diagnostic techniques such as imaging and endomyocardial biopsy. BS for CA is performed using the same tracer in which most oncological pathologies are investigated by CNM in Romania, so it is available in almost all nuclear medicine centers. The brief medical report and the aspect of the images that do not require difficult processing, represent an advantage of the simplicity of the investigation.

Most but not all deposits of cardiac amyloid with mutant and wild-type transthyretin are detected by bisphosphonate scintigraphy. BS for CA can detect fibrillar proteins in the myocardium before the thickening of the heart walls or reduction of voltage on the ECG [16]. From the same kit for the preparation of 99mTc-bisphosphonate, several bone scintigraphy investigations can be made.

### 4.6. The “Romanian” Variant of ATTR Glu54Gln Amyloidosis

The first case of ATTRGlu54Gln amyloidosis was diagnosed in Romania in 2005 by Coriu et al. [31]. So far, more than 20 patients with ATTR Glu54Gln have been diagnosed, all originally from northeastern Romania, Suceava County. The prevalence of ATTRv amyloidosis in Romania is 1.02 per million (0.76 per million for the ATTR Glu54Gln mutation), and in Suceava County, it is 2.39 per 100,000 inhabitants for ATTR-Glu54Gln. The phenotype of patients with ATTRGlu54Gln amyloidosis is mixed (cardiological and neurological), and the clinical onset is in the 4th decade. Jercan et al. reported on the first 18 patients with ATTR Glu54Gln amyloidosis [32]. Of note, scintigraphy showed myocardial radiotracer uptake in all patients, showing high sensitivity of the technique for this mutation. This underlines the importance of improved awareness among NMPs for this diagnostic use of a widespread technique, which can save lives if recommended, performed, and reported properly.

### 4.7. Educational Targets

Looking at the influence of the professional level as a determinant of specific knowledge, we did not find any significant differences between senior, specialist and in training NMPs, which underlines the need for continuing medical education at every step of nuclear medicine career for good usage and interpretation of this technique. Based on our findings and the issues discussed above, we propose that the continuing medical education of NMPs should be continued at all career levels and should focus on the following unmet needs. These include: (1) BS appropriateness (awareness of BS sensitivity and specificity in the positive and differential diagnosis of CA, appropriate indications of BS for CA-ATTR diagnosis); (2) knowledge of protocols (including administered doses, waiting times, recommended acquisition types, use of radiopharmaceuticals recommended in the diagnosis of CA, performing a visual analysis (qualitative) and semi-quantitative (visual score Perugini with uptake grading) in accordance with international recommendations); and (3) multidisciplinary collaboration in CA diagnosis (i.e., the need to inform the referring physician of incidental diagnoses of CA-ATTR, referring the patient to an expert center, imaging correlation with other imaging examinations as part of diagnostic management, establishing multidisciplinary teams specialized in the diagnostic and therapeutic management of the disease, participation in interdisciplinary conferences for the permanent knowledge updating).

## 5. Study Limitation

This survey-based study is small and was designed for nuclear medicine physicians in Romania. The answers of the specialists in nuclear medicine highlighted the current level of knowledge regarding CA. 

One of the limitations of the study is the low addressability, due to the small community of nuclear medicine physicians in Romania, compared to other specialties. However, the respondents exceeded half of the Romanian nuclear physicians’ community from all three levels of training available in Romania. 

The geographical distribution of the respondents was both from the three major university centers in the country, as well as from non-university hospitals or private medical centers. Thus, it was possible to get an overview of knowledge of nuclear medicine physicians in Romania regarding cardiac amyloidosis.

## 6. Conclusions

Knowledge regarding scintigraphy used for CA diagnosis among nuclear physicians in Romania remains one of the biggest challenges. Romanian nuclear physicians are partially familiar with CA diagnosis by scintigraphy, but its diagnostic potential and standardization, recommended radiotracers and acquisition times and interpretation algorithms are known in varying proportions. Therefore, there is a need to enhance knowledge through continuing medical education programs, leading to standardization of the protocols for the acquisition and accurate interpretation of bisphosphonate scintigraphy.

## Data Availability

The data presented in this study are available on request from the corresponding author.

## Supplementary Materials

The copy of the complete online questionnaire is available online at www.mdpi.com/article/10.3390/diagnostics12020556/s1.

## Abbreviations

AA	amyloid fibrils proteins with (Apo) serum amyloid A
AL	amyloid fibrils proteins with light chain
AApoAI	amyloid fibrils proteins with Apolipoprotein A I, variants
AApoAII	amyloid fibrils proteins with Apolipoprotein A II, variants
AApoAIV	amyloid fibrils proteins with Apolipoprotein A IV, variants
Aβ2M	amyloid fibrils proteins with β2-microglobulin
AFib	amyloid fibrils proteins with Fibrinogen α, variants
AGel	amyloid fibrils proteins with Gelasolin variants
ATTR	amyloid fibrils proteins with transthyretin
ATTRv	amyloid fibrils proteins with variant transthyretin
ATTRwt	amyloid fibrils proteins with wild-type transthyretin
BS	bone scan
CA	cardiac amyloidosis
CNM	conventional nuclear medicine
CMR	cardiac magnetic resonance
ECG	electrocardiography
EMB	endomyocardial biopsy
MBq	derived unit for radioactivity
MRI	magnetic resonance imaging
NMPs	nuclear medicine physicians
PET/CT	Positron emission tomography/computed tomography
RNT	radionuclide therapy
SPECT	Single photon-emission computed tomography
SPECT/CT	Single photon-emission computed tomography/computed tomography
TTR	transthyretin
US	ultrasound
