# Supplementary material for: Awareness of Nuclear Medicine Physicians in Romania Regarding the Diagnostic of Cardiac Amyloidosis—A Survey-Based Study
Source: Diagnostics (Basel). 2022 Feb 21;12(2):556. doi: 10.3390/diagnostics12020556 (PMC8870760; doi:10.3390/diagnostics12020556)
Supplement: Supplementary file 1 [file diagnostics-12-00556-s001.zip › diagnostics-1551033-supplementary.pdf]

The perception of Romanian nuclear medicine physicians about bisphosphonate scintigraphy for the detection of cardiac amyloidosis

Dear colleagues,

Thank you for accepting our invitation to participate in this online survey dedicated to nuclear medicine physicians in Romania, witch assessed the curent perception and experience in this field. As we all know, the existence of therapeutic options in cardiac amyloidosis with transthyretin (CA-ATTR) makes it necessary to increase the knowledge of this disease, as well as to make an early diagnosis of ATTR amyloidosis. The survey below includes 31 questions with one or more answers. It takes about 10-15 minutes to complete. Participating in this survey implies your agreement to use the answers to conduct an analysis of your current knowledge and experience with cardiac amyloidosis. The data collected through this survey will be processed completly anonymous. By completing this questionnaire, you agree to voluntarily participate in this question-based research. You can refuse to participate in the study or withdraw at any time before completing the survey. The results will be presented in a research paper.

1. Are you a nuclear medicine phisician?
   a. Yes
   b. No
2. What is the region where you live and work? (droplist)
3. What is your level of experience?
   a. Nuclear Medicine Physician (NMP) in training
   b. Specialized NMP (intermediate level)
   c. Senior NMP (highest level)
4. In what kind of medical center do you work?
   a. Nuclear Medicine Laboratory (public hospital)
   b. Nuclear Medicine Laboratory (private hospital or center)
5. How would you describe the hospital where you worked?
   a. Universitary Hospital
   b. Non-Universitary Hospital
6. How many patients are evaluated by scintigraphy (bone, cardiac, parathyroid, thyroid, etc.), on average, each month?
   a. <50
   b. 51-100
   c. 101-150
   d. 151-200
   e. >200
7. In which subspeciality of nuclear medicine do you practice?
   a. Diagnosis (planar scintigraphy, SPECT or SPECT/CT)
   b. Diagnosis (PET/CT)
   c. Radionuclide therapy (131Iodine, etc.).
8. What types of nuclear cardiology imaging investigations do you perform?
   a. Pefusion myocardial scintigraphy
   b. Bisphosphonate scintigraphy for the detection of cardiac amyloidosis

    c. PET/CT with 18FDG for the detection of infectious endocarditis or vasculitis, etc.
    d. First pass ventriculography
    e. Equilibrium ventriculography
    f. Other
9. Have you attended conferences or courses dedicated to the applications of nuclear medicine in cardiac amyloidosis?
    a. Yes
    b. No
10. Are you involved in the institution where you work in a team dedicated to the diagnosis of cardiac amyloidosis?
    a. Yes
    b. No
11. During your professional activity, did you find a diffuse accumulation of the radiotracer, incidental, in the projection of the heart, during a scintigraphic investigation with bisphosponates performed for another indication than cardiac amyloidosis (for example bone scintigraphy with oncological indication)?
    a. Yes, 1 patient
    b. Yes, between 1-3 patients
    c. Yes, over 3 patients
    d. No.
12. How did you proceed when you detected a diffuse accumulation of the radiotracer, incidental, in the projection of the heart, in a scintigraphic investigation with bisphosphonates?
    a. You noted it as a result without further comment,
    b. You noted this as a result of commenting on the possibility of a diagnosis of cardiac amyloidosis,
    c. You have contacted the sending physician to draw attention to the incidental discovery,
    d. Other
13. What imaging tests are used to evaluate patients with suspected cardiac amyloidosis, in your institution, or in the institution you work with?
    a. Cardiac Ultrasound
    b. Cardiac Magnetic Resonace Imaging
    c. 99mTc-bisphosphonate scintigraphy
    d. Cardiac imaging is recommended to another medical institution
14. 99mTc-labeled bisphospnonate radiotracers used for the detection of cardiac amyloidosis may be:
    a. Hydroxyethylene diphosphonate (HDP)
    b. Hydroxymethylene diphosphonate (HMDP)
    c. 2,3-dicarboxypropane-1,1-diphosphonate (DPD)
    d. Sodium pyrophosphate (PYP)
    e. Methylene diphosphonate (MDP)
15. Detection of myocardial uptake of 99mTc-labeled bisphosphonates on scintigraphy suggests:
    a. Existence of cardiac amyloidosis with tranthyretin (CA-ATTR)
    b. Existence of cardiac amyloidosis with light chain amyloidosis (CA-AL)

c. Existence of uncertain cardiac amyloidosis, and an additional assessement in needed to specify the type of amyloid
d. It is not related to cardiac amyloidosis

16. The semi-quantitative analysis score (Perugini) of 99mTc-bisphosphonate cardiac uptake refers to:
    a. The degree of uptake of the radiotracer from the bone
    b. The degree of uptake of the radiotracer strictly at the level of the costal grid
    c. The degree of uptake of the radiotracer at the level of the heart in relation to the bone uptake
    d. The degree of uptake of the radiotracer at the level of the heart compared to the uptake at the level of the costal arches.
17. Would you be interested in creating teaching materials on systemic amyloidosis and organizing scinetific events in this field?
    a. Yes
    b. No
18. Dou you use bisphosphonate radiotracers to detect cardiac amyloidosis?
    a. Yes
    b. No

    If you are using bisphosphonate radiotracers to detect cardiac amyloidosis, please answer the following 13 questions. Otherwise the questionnaire has ended. Thank you!

19. Would you describe your institution as a reference center for amyloidosis?
    a. Yes
    b. No
20. How are the results of 99mTc-bisphosphonate scintigraphy used in your institution, or in the one you work with? Select the appropriate anwers.
    a. Screening
    b. Diagnosis
    c. Prognosis
    d. Evaluation of the response to therapy
21. Please select the approximate number of bisphosphonate scintigraphic investigations for cardiac amyloidosis that you currently perform in a month:
    a. 0-1
    b. 2-4
    c. 5-10
    d. >10
22. Please enter the average administered dose of 99mTc-bisphosphonate used in scintigraphy for detection of cardiac amyloidosis (in MBq):
    a. <370
    b. 370-554
    c. 555-740
    d. >740
23. Regarding the time period between tha administration of 99mTc-bisphosphonate and the acquisition, the images obtained may be early and late. Please select what you apply in your institution.

a. Early (e.g. 10-15 minutes)
b. Late (e.g. 1-3 hours)

24. Please specify the approximate time period between 99mTc-bisphosphonate administration and early acquisition (in minutes, if applicable).
    a. <10
    b. 10-15
    c. 16-30
    d. 31-45
    e. >45
25. Please choose the approximate time average between 99mTc-bisphosphonate administration and late acquisition (in minutes, if applicable).
    a. 60-89
    b. 90-120
    c. 121-150
    d. 151-180
    e. >180
26. Please select all types of image acquisition that you perform as part of the imaging protocol in the scintigraphic diagnosis of cardiac amyloidosis.
    a. Planar at the level of the thorax
    b. Planar whole body
    c. SPECT at chest level
    d. Cardial SPECT only
27. Dou you use visual (qualitative) analysis of bisphosphonate scintigraphy to describe the appearance of heart uptake (e.g., no uptake, focal uptake, diffuse uptake, focal uptake overlapping diffuze uptake)?
    a. Yes
    b. No
28. How do you report 99mTc-bisphosphonate uptake in the heart in case of scintigraphy for cardiac amyloidosis?
    a. Qualitative (present/absent)
    b. Quantitative or semi-quantitative (various reporting methods)
29. Dou you report the Perugini semi-quantitative visual score of cardiac retention (e.g. VS 0-3)?
    a. Yes
    b. No
30. What diagnostic significance do you assign to visual score Perugini 1 (VS 1)?
    a. It does not represent a diagnostic score and exludes cardiac amyloidosis
    b. It is not necessary to report because it has no clinical significance
    c. Perugini VS 1 makes transthyretin cardiac amyloidosis (CA-ATTR) unlikely
    d. It is diagnosis for light chain amyloidosis (AL).
31. Do you perform the calculation of 99mTc-bisphosphonate uptake from the heart to the contralateral region (H/CL) as part of the interpretation of the scintigraphic examination?
    a. Yes
    b. No.
